# Construction and validation of a first-generation *Bordetella bronchiseptica *long-oligonucleotide microarray by transcriptional profiling the Bvg regulon

**DOI:** 10.1186/1471-2164-8-220

**Published:** 2007-07-06

**Authors:** Tracy L Nicholson

**Affiliations:** 1 Respiratory Diseases of Livestock Research Unit; National Animal Disease Center, USDA, Agricultural Research Service, Ames, Iowa 50010, USA

## Abstract

**Background:**

*Bordetella bronchiseptica *is a bacterial respiratory pathogen that infects a broad range of mammals, causing chronic and often subclinical infections. Gene expression in *Bordetella *is regulated by a two-component sensory transduction system, BvgAS, which controls the expression of a spectrum of phenotypic phases transitioning between a virulent (Bvg^+^) phase and a non-virulent (Bvg^-^) phase.

**Results:**

Based on the genomic sequence and using the freely available software ArrayOligoSelector, a long oligonucleotide *B. bronchiseptica *microarray was designed and assembled. This long-oligonucleotide microarray was subsequently tested and validated by comparing changes in the global expression profiles between *B. bronchiseptica *RB50 and its Bvg^- ^phase-locked derivative, RB54. Data from this microarray analysis revealed 1,668 Bvg-regulated genes, which greatly expands the BvgAS regulon defined in previous reports. For previously reported Bvg-regulated transcripts, the gene expression data presented here is congruent with prior findings. Additionally, quantitative real-time PCR data provided an independent verification of the microarray expression values.

**Conclusion:**

The results presented here provide a comprehensive, genome-wide portrait of transcripts encompassing the BvgAS regulon, while also providing data validating the long-oligonucleotide microarray described here for studying gene expression in *Bordetella bronchiseptica*.

## Background

Bordetellae are Gram negative bacterial respiratory pathogens. *Bordetella pertussis *and *Bordetella parapertussis*_hu_, the causative agents of whooping cough, are human-adapted variants of *Bordetella bronchiseptica*, which naturally infects a broad range of mammals causing chronic and often asymptomatic infections [1]. The majority of gene expression in *Bordetella *is regulated by a two-component sensory transduction system encoded by the *bvg *locus. The *bvg *locus comprises a histidine kinase sensor protein, BvgS, and a DNA-binding response-regulator protein, BvgA. In response to environmental cues, BvgAS controls expression of a spectrum of phenotypic phases transitioning between a virulent (Bvg^+^) phase and a non-virulent (Bvg^-^) phase. During the virulent Bvg^+ ^phase, the BvgAS system is fully active and many of the known virulence factors are expressed, such as filamentous hemagglutinin (FHA), pertactin, fimbriae, adenylate cyclase-hemolysin toxin, and dermonecrotic toxin (DNT), as well as a type III secretion system (TTSS) in *B. bronchiseptica *[2]. Conversely, BvgAS is inactive during the Bvg^- ^phase, resulting in the maximal expression of motility loci, virulence-repressed genes (*vrg *genes), genes required for the production of urease, and in *B. bronchiseptica *RB50, a siderophore, alcaligin [3-5]. Previous studies involving phase-locked and ectopic expression mutants demonstrated that the Bvg^+ ^phase promotes respiratory tract colonization by *B. pertussis *and *B. bronchiseptica *[6-9], while the Bvg^- ^phase of *B. bronchiseptica *promotes survival under conditions of nutrient deprivation [6,10].

The signals that activate BvgAS in nature are unknown. However, in the laboratory, BvgAS is active when the bacteria are grown at 37°C and inactive when grown at temperatures below ~26°C or in medium containing MgSO_4 _or nicotinic acid at concentrations in the millimolar range. Although originally identified as a positive regulator of virulence gene transcription [11], it is now known that BvgAS controls expression of over a hundred different genes whose products are either proven or predicted to participate in a wide variety of cellular activities including many basic physiological functions [12-14]. Additionally, it is now understood that rather than functioning like an ON/OFF switch, BvgAS functions more like a "rheostat" capable of controlling gene expression of many phenotypic phases in response to subtle differences in environmental conditions [10].

The advent of microarray technology has enabled scientists to investigate biological questions, such as those pertaining to bacterial pathogenesis and host-pathogen interactions, in a global fashion. The cDNA microarray represents a popular array type in which double-stranded PCR products are spotted onto glass slides. However, construction of such microarrays presents a number of challenges, largely related to costs associated with amplicon validation, tracking and maintenance. For example, the laborious and problematic tracking of PCR amplicons leads to an estimated 10–30% misidentification [15]. Another limitation of cDNA microarrays is their inability, due to cross-hybridization, to reliably discriminate expression patterns of homologous genes [16]. With oligonucleotide arrays, problems related to clone tracking, homologous gene discrimination, and failed PCR amplicons are avoided, thus making long-oligonucleotide microarrays a more cost- and management- efficient alternative to cDNA microarrays. Here we present the design and assembly of a long-oligonucleotide *B. bronchiseptica *gene-specific microarray using the currently available genomic sequence generated by the Sanger Institute [17] and the software package ArrayOligoSelector [18]. This long-oligonucleotide microarray was then tested and validated by evaluating changes in the global expression profiles between *B. bronchiseptica *strain RB50 and its Bvg^- ^phase-locked derivative, RB54.

## Results and discussion

To construct a *B. bronchiseptica*-specific whole genome microarray, the freely available software program, ArrayOligoSelector (AOS) [18], was used to generate 70-mer oligonucleotide probes for every ORF in the *Bordetella bronchiseptica *RB50 genome [17]. The rationale behind designing and utilizing oligonucleotide probes versus PCR amplicons as probes, and subsequently the 70-mer length of the oligonucleotide probes, was chosen for several reasons. Long oligonucleotides are a highly sensitive alternative to PCR products and provide a means to readily distinguish between genes with high degrees of sequence similarity, which is an issue for the *B. bronchiseptica *genome [17]. For example, except for the extreme 5' and 3' termini, the *fhaB *and *fhaS *genes are nearly identical [19]. Additionally, previous results involving an anchored set of oligonucleotides revealed a strong relationship between the oligonucleotide length and hybridization performance [18].

For each *B. bronchiseptica *ORF, the AOS program optimizes the oligonucleotide selection on the basis of uniqueness in the genome, sequence complexity, lack of self-binding and GC content. Candidate oligos closest to the 3' end of the gene are then chosen [18]. There are a number of missing array elements due to gene duplications, prophage duplications, and ORF assignments missing from the completed genome annotation [17]. These missing array elements and, in the case of gene duplications, their corresponding represented array elements are listed in Additional File 1- see Supplementary Table S1. A list of the final 4975 oligonucleotide array elements or probes representing the entire *B. bronchiseptica *genome is given in Additional File 1- see Supplementary Table S2.

To test the usefulness of the newly constructed long-oligonucleotide *Bordetella bronchiseptica *microarray, a direct comparison between the transcriptional profile of *B. bronchiseptica *RB50 and RB54, a *B. bronchiseptica *Bvg^- ^phase locked derivative of RB50, was performed. The rationale behind performing this comparison to validate and test the *B. bronchiseptica *long-oligonucleotide microarray is that this comparison will globally identify *B. bronchiseptica *genes regulated by BvgAS. Previous studies, including cDNA microarray studies, have identified 538 genes controlled by BvgAS [2,13,14], thus providing a substantial reportable reference set to validate gene expression data generated from the newly constructed *B. bronchiseptica *long-oligonucleotide microarray.

**Figure 1:**
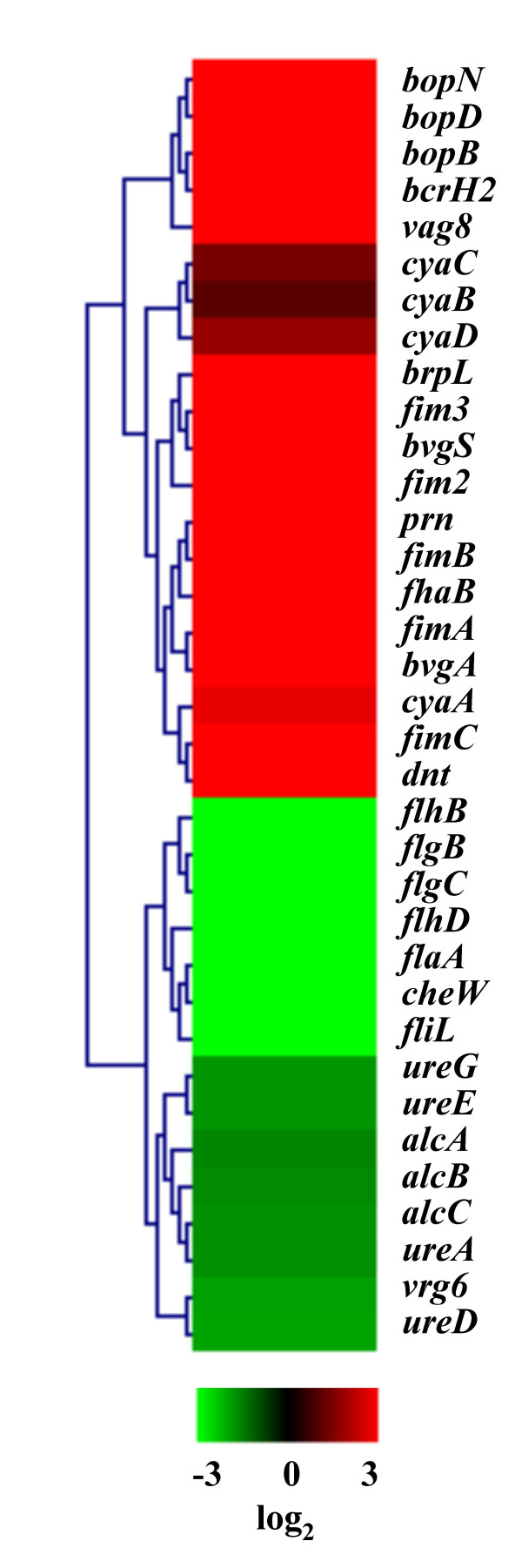
**Expression of demonstrated Bvg-regulated genes**. Hierarchical clustering of known *B. bronchiseptica *Bvg-regulated genes performed using MeV [32]. Expression profiles of genes are in rows. Data are mean centered for each array element and averaged from biological replicates. Red, indicates increased expression in RB50; green, decreased gene expression in RB50 (and increased expression in RB54); black, no significant change in gene expression.

Utilizing the *B. bronchiseptica *long-oligonucleotide microarray, ratio data collected from microarray analysis involving the direct comparison between *B. bronchiseptica *RB50 and RB54 revealed 1,668 Bvg-regulated genes (identified using SAM with a false discovery rate of <0.1%). This is a substantial increase in the number of Bvg-regulated transcripts compared to the 538 Bvg-regulated genes recently reported by others using cDNA microarray analysis [14]. A complete list of the fold-change expression values from this comparison, along with dye swap experimental data, is provided in Additional File 1- Supplementary Table S3. One possible effect of using the *B. bronchiseptica *RB50 strain grown at 37°C to represent the Bvg^+ ^phase, as opposed to using a Bvg^+ ^phase-locked strain, is that some Bvg-activated genes could be missed in this analysis. Possible explanations for the large increase in Bvg-regulated transcripts detected in this study, compared to previous reports, may include (i) differences between strains used, for example wild-type versus phase-locked derivatives, (ii) differences between gene expression threshold cut-off values used in analysis, and (iii) for microarray studies, differences between array platforms, such as cDNA microarrays versus oligonucleotide microarrays. Gene expression profiles reported here of demonstrated Bvg-regulated transcripts are consistent with previous results (Figure 1).

Historically, numerous Bvg-activated genes have been described, such as TTSS genes, fimbrial genes, filamentous hemagglutinin, pertactin, adenylate cyclase-hemolysin toxin, and dermonecrotic toxin [2]. Using the newly constructed oligonucleotide *B. bronchiseptica *microarray, all of these transcripts were found to be positively regulated by BvgAS (Figure 1). Along with other virulence-related transcripts classically characterized as Bvg-activated, three putative adhesion genes *fhaS *(+10.37-fold), *fhaL *(+8.04-fold), and BB0110 (+8.58-fold) were found to be highly Bvg-activated, as well as a putative novel toxin, BB3242 (+50.72-fold), containing an aerolysin and pertussis toxin domain (Table 1 and see Additional File 1- Supplementary Table S3). Seven ATP-binding cassette (ABC) transporters were found to be Bvg-activated, including BB1593 (+83-fold) (Table 1 and see Additional File 1- Supplementary Table S3). In other bacteria, these transporters serve as important virulence factors based upon their role in nutrient uptake and in secretion of toxins and antimicrobial agents [20]. Five autotransporters were highly Bvg-activated, including two virulence-associated transcripts *vag8 *and *sphB1 *(Table 1 and see Additional File 1- Supplementary Table S3). The autotransporter *sphB1 *has been implicated in the maturation process of FHA [21]. The Bvg-activated expression profile of these transporters implicates their importance in pathogenesis. Five genes involved in protein folding and ushering were highly Bvg-activated. These include *dsbG *(+35.29-fold) and *mucD *(+2.75-fold), a DegP family serine protease (Table 1 and see Additional File 1- Supplementary Table 3). Homologues of both *dsbG *and *mucD *are known to serve roles in virulence in other organisms [22,23]. Twenty-seven transcripts identified as serving roles in global regulatory functions were found to be Bvg-activated, including the conventionally characterized *bvgAS *and the TTSS regulator *brpL *(Table 1 and see Additional File 1- Supplementary Table S3). Other Bvg-activated transcripts in this group include the other TTSS regulators (BB1645, BB1646, and BB1642), three proposed two-component response regulatory proteins, and numerous proposed transcriptional regulators. Lastly, BB4228 (+15.70-fold), recently designated BopC and characterized as a novel type-III secretion effector [24], was found to be highly Bvg-activated.

**Table 1:** Representative Bvg^+ ^phase specific genes identified by microarray analysis.

**Function**	**Gene**	**Product**	**Fold-Change ± SEM**
Cell Adhesin	BB0110	putative adhesion	8.58 ± 3.21
	BB1658 *fim3*	serotype 3 fimbrial subunit precursor	32.47 ± 11.10
	BB1936 *fhaL*	adhesion	8.04 ± 1.63
	BB2312 *fhaS*	adhesion	10.37 ± 2.76
	BB2989 *fimD*	fimbrial adhesion	11.58 ± 3.96
	BB2992 *fimA*	fimbrial protein	33.75 ± 13.88
	BB2993 *fhaB*	filamentous hemagglutin	11.37 ± 4.55
	BB3424	fimbrial protein	8.92 ± 2.64
	BB3425 *fimN*	fimbrial subunit protein	11.95 ± 2.06
	BB3426 *fimX*	fimbrial protein	8.92 ± 2.64
	BB3674 *fim2*	serotype 2 fimbrial subunit precursor	21.89 ± 11.68
Cell Envelope	BB1289	putative integral membrane protein	26.29 ± 8.25
	BB1368	putative membrane protein	3.94 ± 0.56
	BB3119	putative membrane protein	19.27 ± 2.17
	BB3157	putative membrane protein	16.41 ± 5.54
	BB4029	putative glycosyl transferase	4.96 ± 0.32
	BB4266	putative membrane protein	6.84 ± 0.82
	BB4269	putative glycosyl transferase	2.23 ± 0.42
	BB4284	putative membrane protein	2.96 ± 0.14
Protein Folding and Ushering	BB2320 *dsbG*	thiol:disulfide interchange protein precursor	35.29 ± 9.68
	BB2991 *fimB*	chaperone protein	11.20 ± 7.71
	BB3749 *mucD*	serine protease; trypsin activity	2.75 ± 0.41
	BB3803	putative peptidyl-prolyl cis-trans isomerase	4.65 ± 1.26
Global Regulatory Functions	BB0308	putative transcriptional regulator	5.67 ± 0.98
	BB1607	putative LysR-family transcriptional regulator	3.37 ± 0.55
	BB1638 *brpL*	putative RNA polymerase sigma factor	50.66 ± 15.58
	BB1642	putative regulator; catalytic activity	112.16 ± 97.97
	BB1645	putative anti-sigma factor; ATP binding	9.46 ± 3.00
	BB1646	putative antisigma factor antagonist	18.19 ± 6.08
	BB2051	putative regulatory protein	2.24 ± 0.59
	BB4374	putative AsnC-family transcriptional regulator	5.78 ± 7.25
Metabolism	BB0457	probable enoyl-CoA hydrates; isomerase activity	5.72 ± 1.34
	BB0458	probable carboxymuconolactone decarboxylase	3.42 ± 0.79
	BB1476	putative gluconate dehydrogenase	2.88 ± 0.51
	BB1478	putative carbonic anhydrase precursor	4.05 ± 0.77
	BB1640	hypothetical protein; glutamate-cysteine ligase activity	63.92 ± 18.15
	BB1644 *alr*	alanine racemase, catabolic	253.96 ± 97.47
	BB2658	glutamate decarboxylase	2.78 ± 0.93
	BB3783	intracellular PHB depolymerase	3.23 ± 0.78
	BB4286 *nadC*	putative nicotinate-nucleotidepyrophosphorylase	32.86 ± 6.48
Periplasmic, Exported, Lipoprotein	BB0324 *cyaA*	bifunctional hemolysin-adenylatecyclase precursor	6.73 ± 2.52
	BB1280	putative exported protein	73.44 ± 37.80
	BB1292	putative exported protein	272.88 ± 93.88
	BB1641	putative exported protein	149.05 ± 97.51
	BB2398	putative exported protein	6.77 ± 1.38
	BB2873	putative membrane protein	26.85 ± 13.90
	BB3068	putative exported protein	10.90 ± 3.11
	BB3242	putative exported protein	48.75 ± 9.65
	BB3302	lipoprotein	5.09 ± 0.63
	BB3978 *dnt*	dermonecrotic toxin	8.63 ± 3.61
	BB4181	putative lipoprotein	3.40 ± 0.25
	BB4285	putative exported protein	49.73 ± 14.04
Transport	BB0323 *cyaC*	cyclolysin-activating lysine-acyltransferase	2.70 ± 0.27
	BB0325 *cyaB*	cyclolysin secretion ATP-binding protein; ABC transporter	2.10 ± 0.31
	BB0326 *cyaD*	cyclolysin secretion protein; transporter activity	3.48 ± 0.51
	BB0327 *cyaE*	cyclolysin secretion protein; subtilase activity	3.66 ± 1.57
	BB0419 *sphB1*	autotransporter subtilisn-like protease; substilase activity	59.34 ± 43.26
	BB1593	putative ABC transport system, membrane protein	83.60 ± 28.88
	BB1643	putative inner membrane transport protein	39.61 ± 16.68
	BB1864 *vag8*	autotransporter	122.00 ± 79.14
	BB2270	autotransporter	4.71 ± 2.07
	BB3826 *bfrD*	probable TonB-dependent receptor for iron transport	2.68 ± 0.58
	BB3993 *ompQ*	outer membrane porin protein OmpQ	24.34 ± 8.07
TTSS	BB1616 *bopN*	putative outer protein N	504.05 ± 189.58
	BB1620 *bopD*	putative outer protein D	457.16 ± 172.40
	BB1624 *bscI*	putative type III secretion protein	226.35 ± 80.58
	BB1628 *bscN*	putative ATP synthase in type III secretion	115.85 ± 37.00
	BB4228 *bopC*^a^	hypothetical protein; characterized type III effector	15.70 ± 3.85

DNA microarray analysis was used to measure mRNA levels present in *B. bronchiseptica *RB50 compared to mRNA levels present in *B. bronchiseptica *RB54. Differences in mRNA levels are listed as mean fold-changes ± standard error. Fold-changes were calculated by averaging the data from three biological sample sets. ^a^Recently characterized [24].

The overall global transcriptional program observed during the Bvg^- ^phase supports previous data implicating a crucial role for the Bvg^-^phase in survival during environmental stress conditions [2,25]. Genes annotated to serve global regulatory functions were one of the functional categories with the highest number of Bvg-repressed transcripts. Transcripts in this category include forty-nine transcription factors and/or DNA-binding proteins, and ten two-component systems (Table 2 and see Additional File 1- Supplementary Table 3). Numerous genes involved in transport were identified as Bvg-repressed, including twenty-seven ABC transporters and TonB-dependent receptors, such as *bfrG *BB1294 (-2.86-fold) and *bfeA *BB1942 (-2.80-fold) (Table 2 and see Additional File 1- Supplementary Table S3). As mentioned earlier, these transporters serve important biological roles in other organisms, including participation in host-pathogen interactions as known virulence factors [20]. The Bvg-repressed expression profile of these genes highlights nutrient scavenging as a critical ability during Bvg^- ^growth and supports prior data demonstrating that the Bvg^- ^phase is optimized for growth under nutrient limiting conditions [2,25].

**Table 2:** Representative Bvg^- ^phase specific genes identified by microarray analysis.

**Function**	**Gene**	**Product**	**Fold-Change ± SEM**
Cell Adhesin	BB1186	putative hemolysin, homophilic cell adhesion	-2.83 ± 0.18
Cell Envelope	BB0141 *wbmD*	putative membrane protein	-5.19 ± 0.08
	BB2618 *flgJ*	peptidoglycan hydrolase	-28.12 ± 0.01
	BB2618 *lpxB*	lipid-A-disaccharide synthase; lipid A biosynthesis	-6.35 ± 0.02
	BB2879	putative membrane protein	-6.46 ± 0.02
	BB4842	putative outer membrane protein	-32.02 ± 0.01
Chemotaxis	BB2543 *motA*	chemotaxis protein MotA	-26.28 ± 0.02
	BB2544 *motB*	chemotaxis protein MotB	-17.90 ± 0.02
	BB2549 *cheR*	chemotaxis protein methyltransferase	-10.43 ± 0.03
	BB2552 *cheZ*	chemotaxis protein CheZ; catalytic activity	-38.44 ± 0.01
Motility	BB2553 *flhB*	flagellar biosynthetic protein FlhB	-44.48 ± 0.01
	BB2555 *flhF*	flagellar biosynthesis protein; RNA binding	-32.10 ± 0.00
	BB2559 *flgB*	flagellar basal-body rod protein FlgB; motor activity	-44.49 ± 0.01
	BB2560 *flgC*	flagellar basal-body rod protein FlgC; motor activity	-32.10 ± 0.00
	BB2585 *fliI*	flagellum-specific ATP synthase FliI; ATP binding	-30.75 ± 0.01
Electron Transport	BB1283 *cyoA*	ubiquinol oxidase polypeptide II; copper ion binding	-5.11 ± 0.08
	BB2800 *napA*	periplasmic nitrate reductase precursor; iron ion binding	-11.34 ± 0.01
	BB3325	putative ferredoxin; electron transport activity	-32.43 ± 0.00
	BB3927	putative cytochrome; electron transport activity	-5.58 ± 0.01
	BB4096	putative oxidoreductase	-4.90 ± 0.02
	BB4497 *cydB*	cytochrome D ubiquinol oxidase subunit II	-6.04 ± 0.02
	BB4498 *cydA*	cytochrome D ubiquinol oxidase subunit I	-5.40 ± 0.07
	BB4945 *ivd*	isovaleryl-CoA dehydrogenase	-8.90 ± 0.01
Protein Folding and Ushering	BB0979	putative universal stress protein	-24.76 ± 0.02
	BB2875	universal stress family protein	-7.45 ± 0.01
	BB3257 *fkpB*	FkbP-type peptidyl-prolyl cis-trans isomerase	-4.15 ± 0.01
	BB4260	putative universal stress protein	-9.29 ± 0.01
Global Regulatory Functions	BB0725	putative transcriptional regulator	-7.82 ± 0.04
	BB1122	two-component system sensor kinase; signal transducer	-9.65 ± 0.01
	BB1187	putative LuxR-family transcriptional regulator	-11.60 ± 0.02
	BB2108	probable two-component response regulator	-18.08 ± 0.01
	BB2323	putative transcriptional regulator	-11.03 ± 0.03
	BB2540 *fliA*	RNA polymerase sigma factor; flagellar operon	-16.05 ± 0.01
	BB2542 *flhC*	flagellar transcriptional activator FlhC; DNA binding	-31.06 ± 0.01
	BB2550 *cheB*	protein-glutamate methylesterase;two-component regulator	-18.56 ± 0.02
	BB3115	methyl-accepting chemotaxis signal transducer protein	-10.74 ± 0.02
	BB3866	probable LysR-family transcriptional regulator	-7.15 ± 0.02
Metabolism	BB0868 *mmsA*	methylmalonate-semialdehyde dehydrogenase	-8.72 ± 0.02
	BB0978 *ggt*	gamma-glutamyltranspeptidase precursor	-35.18 ± 0.02
	BB1367 *cysG*	siroheme synthase, heme biosynthesis	-8.67 ± 0.01
	BB2085 *hemC*	porphobilinogen deaminase; synthase activity	-16.39 ± 0.01
	BB2147 *hemN*	coproporphyrinogen III oxidase	-5.84 ± 0.01
	BB4409 *hemL*	glutamate-1-semialdehyde 2,1-aminomutase	-13.10 ± 0.01
Transport	BB0976	lactate permease family protein	-21.29 ± 0.01
	BB1174	putative ABC transport proteins; ATP-binding component	-5.52 ± 0.03
	BB1188	HlyD-family secretion protein; protein transporter activity	-6.27 ± 0.05
	BB1189	probable ABC transporter	-7.56 ± 0.06
	BB1191	putative outer membrane protein; transporter activity	-13.87 ± 0.02
	BB1294 *bfrG*	putative TonB-dependent receptor	-2.86 ± 0.03
	BB1942 *bfeA*	ferric enterobactin receptor	-2.80 ± 0.02
	BB2402	putative sulfate transporter	-6.92 ± 0.02
	BB2433	multidrug resistance protein	-4.14 ± 0.03
	BB2803 *ccmA*	putative heme export protein; heme transporter activity	-12.62 ± 0.01
	BB2804 *ccmB*	heme exporter protein B	-9.57 ± 0.01
	BB2805 *ccmC*	putative heme export protein	-13.01 ± 0.01
	BB4273 *atoE*	putative short-chain fatty acids transporter	-7.49 ± 0.01
	BB4495	probable ATP-binding component; ABC transporter	-33.13 ± 0.01

DNA microarray analysis was used to measure mRNA levels present in *B. bronchiseptica *RB50 compared to mRNA levels present in *B. bronchiseptica *RB54. Differences in mRNA levels are listed as mean fold-changes ± standard error. Fold-changes were calculated by averaging the data from three biological sample sets.

Consistent with previous results, genes required for the expression of the siderophore alcaligin [26] and urease [5] were preferentially activated in the Bvg^- ^phase, along with a putative hemolysin BB1186 (-2.83-fold) (Table 2 and see Additional File 1- Supplementary Table S3). Also consistent with previous reports, all of the genes known to be involved in chemotaxis, such as *cheD *(-10.59-fold) and *cheW *(-15.21-fold), and motility, such as *flgB *(-44.49-fold) and *flgC *(-32.10-fold), were Bvg-repressed [3] (Table 2 and see Additional File 1- Supplementary Table S3). Numerous genes involved in electron transport were Bvg-repressed along with numerous genes involved in protein folding and ushering, including a high number of universal stress proteins, congruent with the Bvg^- ^phase role in stress survival [6,25] (Table 2 and see Additional File S1- Supplementary Table S3).

*Bordetella pertussis *only infects humans and, as mentioned above, is responsible for causing an acute upper-respiratory disease known as whooping cough or pertussis. Due to its high degree of similarity to *B. pertussis *and its broad host range, including animals conveniently used in laboratory studies, *B. bronchiseptica *is widely used as a model for *Bordetella *pathogenesis research. Data arising from the recent completion of the comparative sequencing of three different *Bordetella *strains has revealed a loss or inactivation of a large number of genes in *B. pertussis *[17]. Thus an intriguing question is how many of the *B. bronchiseptica *Bvg-regulated genes are intact in *B. pertussis*. To determine this, the ORF sequence and oligonucleotide probe sequence for every Bvg-regulated gene identified in this study was used to blast the *B. pertussis *genome sequence. This analysis resulted in identifying 1172 shared genes that are Bvg-regulated in *B. bronchiseptica *(see Additional File S1- Supplementary Table S4).

**Figure 2:**
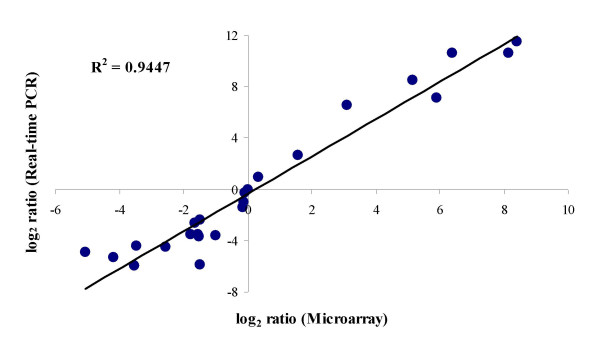
**Comparison of gene expression measurements by microarray hybridization and quantitative real-time PCR**. Changes in gene expression were log transformed (in base 2), and the real-time qRT-PCR log_2 _values (y axis) were plotted against the microarray data log_2 _values (x axis). The coefficient of determination (R^2^) is given.

Real-time quantitative PCR was performed to provide an independent assessment of microarray expression measurements for selected genes. Genes were chosen to reflect the full spectrum of fold-changes identified by microarray analysis. Specifically, transcripts identified as having little to no change in gene expression by microarray analysis and selected for qRT-PCR include BB0057 *rpoA*, BB4989 *dnaA*, BB1037, and BB3703 *eno *(Table 3). Bvg-activated genes identified by microarray analysis and selected for qRT-PCR include the TTSS regulatory genes, BB1619 *bcrH1 *and BB1622 *bcrH2 *(Table 3). On the polar end of the Bvg spectrum, genes identified as Bvg-repressed and selected for qRT-PCR include BB2522 and BB1315, a putative universal stress protein. Additionally, BB4835 *rpoH*, sigma-32, identified as Bvg-repressed (-3.09-fold) was also selected for qRT-PCR analysis since it had not been identified as Bvg-regulated by previous cDNA microarray studies [13,14]. Real-time quantitative PCR analysis of this gene set provided data consistent with the quantitative measures by microarray analysis, using the newly constructed *B. bronchiseptica *oligonucleotide microarray (r^2 ^= 0.94) (Figure 2). Therefore, this real-time quantitative PCR data provides independent verification of the microarray results.

**Table 3:** Fold-changes identified by quantitative real-time PCR.

**Gene**	**Product**	**qRT-PCR**	**Microarray**
BB0057 *rpoA*	DNA-directed RNA polymerase alpha chain	-2.03 ± 0.01	-1.09 ± 0.06
BB0110	putative adhesion	92.47 ± 0.35	8.58 ± 1.21
BB0376	putative membrane protein	6.23 ± 0.45	3.00 ± 0.72
BB0419 *sphB1*	autotransporter subtilisin-like protease	137.34 ± 6.96	59.34 ± 4.26
BB1037	LysR-family transcriptional regulator	-1.24 ± 0.01	-1.07 ± 0.15
BB1315	putative universal stress protein	-64.15 ± 0.35	-11.43 ± 0.03
BB1593	putative ABC transport system, membrane protein	1544.59 ± 8.49	83.60 ± 8.88
BB1619 *bcrH1*	putative regulatory protein	2943.53 ± 16.33	340.10 ± 7.39
BB1622 *bcrH2*	putative regulatory protein	1532.48 ± 11.25	286.50 ± 9.03
BB2108	probable two-component response regulator	-39.21 ± 1.24	-18.08 ± 0.01
BB2147 *hemN*	oxygen-independent coproporphyrinogen III oxidase	-23.10 ± 3.33	-5.84 ± 0.01
BB2320 *dsbG*	thiol:disulfide interchange protein precursor	358.29 ± 5.25	35.29 ± 1.68
BB2323	putative transcriptional regulator	-21.86 ± 3.46	-11.03 ± 0.01
BB2367	putative regulatory protein; transcription factor activity	-11.71 ± 2.15	-2.95 ± 0.11
BB2434 *pckG*	phosphoenolpyruvate carboxykinase (GTP)	-12.57 ± 1.46	-2.88 ± 0.04
BB2435	S-adenosylmethionine-dependent methyltransferase	-5.10 ± 1.24	-2.76 ± 0.04
BB2522	conserved hypothetical protein	-58.35 ± 0.35	-2.82 ± 0.03
BB3447 *cysN*	sulfate adenylyltransferase subunit 1; (ATP) activity	-12.50 ± 0.15	-2.00 ± 0.25
BB3703 *eno*	enolase; phosphopyruvate hydratase activity	-2.68 ± 0.14	-1.11 ± 0.08
BB4495	probable ATP-binding component of ABC-transporter	-30.63 ± 0.15	-33.12 ± 0.01
BB4506 *rpoN*	probable sigma (54) modulation protein	-11.66 ± 0.56	-3.38 ± 0.12
BB4835 *rpoH*	RNA polymerase sigma-32 factor	-6.33 ± 0.22	-3.09 ± 0.11
BB4989 *dnaA*	chromosomal replication initiator protein	1.94 ± 0.01	1.27 ± 0.19

Quantitative real-time PCR analysis was used to measure mRNA levels present in *B. bronchiseptica *RB50 compared to mRNA levels present in *B. bronchiseptica *RB54. Differences in mRNA levels detected by both qRT-PCR and Microarray are listed as mean fold-changes ± standard deviation. qRT-PCR fold-changes were calculated using the relative quantitative (ΔΔC_T_) method, as detailed in the Methods, from three biological sample sets.

Given that BvgAS is the major virulence regulator in *Bordetella*, the information presented in this report should allow researchers to design future experiments targeting some newly identified Bvg-regulated genes and study their role in the pathogenesis of this organism. A recent study demonstrated that *Bordetella bronchiseptica*, *Bordetella parapertussis *and *Bordetella pertussis *all contain a higher number of transcription factors or regulatory elements than the genome size of each strain would predict [27]. The microarray analysis presented here identifies, for the first time, an additional 138 predicted global regulator elements as being a part of the BvgAS regulon, which could be described as a modulon. Moreover, the data indicates that a high number of *B. bronchiseptica *regulators are under control, either directly or indirectly, of BvgAS, thus highlighting an organized hierarchical network, with multiple layers of control, governing transcriptional regulation in *B. bronchiseptica*. Distinct phenotypic changes occurring at each end of the Bvg regulatory continuum have been described since 1960 [28]. Since then, the molecular basis underlying these morphological phases has been directly linked to the coordinate expression of the BvgAS regulon. The results presented here provide a comprehensive, genome-wide portrait of transcripts encompassing the BvgAS regulon, while also providing data validating the usefulness of utilizing the long-oligonucleotide microarray described here for studying gene expression in *B. bronchiseptica*.

## Conclusion

Long-oligonucleotide microarrays represent a highly sensitive, cost and management efficient, alternative to cDNA microarrays. Using the complete *Bordetella bronchiseptica *genome sequence and ArrayOligoSelector software, a 70-mer oligonucleotide *Bordetella bronchiseptica *microarray was designed and assembled. This long-oligonucleotide microarray was then tested and validated by comparing changes in the global expression profiles between *Bordetella bronchiseptica *RB50 and its Bvg^- ^phase-locked derivative, RB54. Data from this microarray analysis revealed 1,668 Bvg-regulated genes, which dramatically increases the number of Bvg-regulated transcripts identified to date. Additionally, gene expression profiles of previously demonstrated Bvg-regulated transcripts are consistent with previous results and quantitative real-time PCR data provided an independent verification of the microarray expression values. The results presented here provide a comprehensive, genome-wide portrait of transcripts encompassing the BvgAS regulon, and provide data validating the use of the long-oligonucleotide microarray described here for studying gene expression in *Bordetella bronchiseptica*.

## Methods

### Microarray fabrication

Using ArrayOligoSelector [18], one 70-mer oligonucleotide probe was selected to represent each *B. bronchiseptica *RB50 ORF [17]. The 70-bp oligonucleotide probes were synthesized (Illumina, San Diego, CA), resuspended in 3×SSC to a final concentration of 60 pmol/μL, and spotted onto poly-L-lysine-coated microscope slides, using a BioRobotics Microgrid II microarrayer (Genomic Solutions, Ann Arbor, MI) as described elsewhere [29]. Each probe was printed three times per slide. All oligonucleotide sequences are listed (see Additional File 1- Supplementary Table S2).

### *Bordetella *Culture and RNA Isolation

*B. bronchiseptica *strain RB50 was isolated from a naturally infected New Zealand White rabbit [6] and RB54 is a Bvg^- ^phase-locked derivative of RB50 [10]. *B. bronchiseptica *strains were maintained on Bordet-Gengou (BG) agar (Difco) containing 10% defibrinated sheep blood for determination of colony morphology and hemolytic activity. Three independent biological replicates of *B. bronchiseptica *RB50 and RB54, picked from well-isolated colonies on BG plates, were initially grown in Stainer-Scholte (SS) broth [30] supplemented with 40 μg/mL streptomycin. To ensure similar inocula and growth phase among all biological replicates, bacteria were then subcultured at a starting optical density at 600 nm (OD_600_) of 0.02 into a 250-mL flask containing 50 mL SS broth and grown at 37°C with shaking at 275 rpm for 24 hours. At this time the OD_600 _for the RB50 cultures were approximately 1 and the OD_600 _for the RB54 cultures were approximately 3.5. Total cellular RNA was extracted using Trizol reagent (Invitrogen, Carlsbad, CA), treated with RNase-free DNase I (Invitrogen, Carlsbad, CA), and purified using RNeasy columns (Qiagen, Valencia, CA) according to manufacturers' recommended protocols.

### Preparation of labeled cDNA

A 2-color hybridization format was used for the microarray analysis. For each biological replicate, RNA extracted from *B. bronchiseptica *RB50 was used to create Cy5-labeled cDNA and RNA extracted from *B. bronchiseptica *RB54 was used to create Cy3-labeled cDNA. Conversely, dye-swap experiments were performed analogously, in which RNA extracted from *B. bronchiseptica *RB50 was used to create Cy3- labeled cDNA and *B. bronchiseptica *RB54 was used to create Cy5- labeled cDNA. Fluorescently-labeled cDNA copies of the total RNA pool were prepared by direct incorporation of fluorescent nucleotide analogs during a first-strand reverse transcription (RT) reaction as follows: 5 μg total RNA and 4.4 μg of random oligonucleotide hexamers were incubated 2 minutes at 98°C, cooled on ice, combined with SuperScript III RTase buffer, 0.5 mM dATP, dGTP, dCTP, 0.2 mM dTTP, 1.5 nmol Cy3- or Cy5-dUTP (Amersham), and 2 μL SuperScript III RTase (in a volume of 26 μL). This mixture was incubated 10 minutes at 25°C and 120 minutes at 50°C. The two differentially labeled reactions to be compared were mixed and buffer exchange, purification, and concentration was accomplished by microcon-10 (Amicon) filtration.

### Microarray hybridization and data analysis

Oligonucleotide microarrays were first prehybridized for 1 hour in 5×SSC, 1% BSA and 0.1% SDS at 42°C, followed by washing with H_2_O and then with isopropanol and dried by centrifugation for 5 minutes at 50×g. Following prehybridization, 45 μL hybridization solution (labeled cDNA, 5 μg tRNA, 2×SSC, 25% formamide, 0.1% SDS) was applied to oligonucleotide microarrays and incubated in a humidified chamber overnight at 50°C. Arrays were subsequently removed from humidified chambers and quickly submerged and washed in 1×SSC and 0.05% SDS for approximately 2 minutes, followed by two additional washes for 2 minutes each in fresh 0.06×SSC. Slides were then dried by centrifugation for 5 minutes at 50×g, scanned using a GenePix 4000B microarray scanner, and analyzed with GenePix Pro software (Axon Instruments, Union City, CA). Spots were assessed visually to identify those of low quality and arrays were normalized so that the median of ratio across each array was equal to 1.0. Automatically and manually flagged spots, spots with the sum of medians (635/532) signal intensity less than or equal to 100, and spots with signal intensity below threshold (sum of median intensities plus one standard deviation above the mean background) were filtered out prior to analysis. Ratio data from the three biological replicates were compiled and normalized based on the total Cy3% intensity and Cy5% intensity to eliminate slide to slide variation. Gene expression data were then normalized to 16S rRNA. The statistical significance of the gene expression changes observed was assessed by using the significant analysis of microarrays (SAM) program [31]. A one-class unpaired SAM analysis using a false discovery rate of 0.063% (<0.1%) was preformed. Hierarchical clustering of microarray data using Euclidean Distance metrics and Average Linkage clustering was performed using MeV software from TIGR [32].

### Quantitative real-time PCR

DNase-treated total RNA (1 μg) from each biological replicate was reverse transcribed using 300 ng of random oligonucleotide hexamers and SuperScript III RTase (Invitrogen, Carlsbad, CA) according to manufacturer's protocol. The resulting cDNA was diluted 1:1000 and 1 μL used in quantitative PCR reactions containing 300 nM primers and 2XSYBR Green PCR Master Mix (Applied Biosystems) using an Applied Biosystems 7300 real-time PCR detection system (Applied Biosystems, Foster City, CA). Primers were designed using Primer Express software (Applied Biosystems, Foster City, CA) and are listed in Additional File 1- Supplementary Table 4. To confirm the lack of DNA contamination, reactions without reverse transcriptase were performed. Dissociation curve analysis was performed for verification of product homogeneity. Threshold fluorescence was established within the geometric phase of exponential amplification and the cycle of threshold (Ct) determined for each reaction. The cycle of threshold (Ct) from all three biological replicates for each strain (RB50 and RB54) was compiled and the 16S RNA amplicon was used as an internal control for data normalization. Fold change in transcript level was determined using the relative quantitative method (ΔΔC_T_) [33].

### Microarray accession numbers

Microarrays have been deposited in ArrayExpress under accession number E-MEXP-961.

## Authors' contributions

TLN performed *B. bronchiseptica *culturing, microarray design, microarray construction, microarray analysis, cluster analysis, quantitative real-time PCR analysis, data analysis, and drafted the manuscript. TLN read and approved the final manuscript.

## Supplementary Material

Additional file 1 **Table S1. Missing array elements due to gene duplications, prophage duplications, and ORF assignments missing from the completed genome annotation**. In the Gene Duplication category, the ORF number representing the array element for both genes is given. **Table S2. Oligonucleotide sequence for each ORF/represented array element**. **Table S3. Fold-Change expression values from a direct comparison between the transcriptional profile of *B. bronchiseptica *RB50 and RB54**. DNA microarray analysis was used to measure mRNA levels present in *B. bronchiseptica *RB50 compared to mRNA levels present in *B. bronchiseptica *RB54. Differences in mRNA levels are listed as mean fold-changes + standard error. Fold-changes were calculated by averaging the data from three biological sample sets. The fluorescent labels were exchanged in dye-swap experiments performed on all three biological replicates. ORF, Name, Product, Function, and General Category headings were parsed from both Sanger annotation files [17] and Cummings et al. [14]. Data presented in the SAM, Score(d), q-value, and localdr(%) columns were assessed by using the significant analysis of microarrays (SAM) program [31]. A one-class unpaired SAM analysis using a false discovery rate of 0.063% (<0.1%) was preformed. **Table S4. Quantitative real-time PCR primers**. Click here for file
